# Associations Between Cutaneous Melanoma Traits and the Likelihood of Metastasis

**DOI:** 10.7759/cureus.65754

**Published:** 2024-07-30

**Authors:** Grinvydas Butrimas, Robertina Cibulskaitė, Ugnė Janonytė, Rūta Silickytė

**Affiliations:** 1 Dermatology, Lithuanian University of Health Sciences (LSMU), Kaunas, LTU; 2 Gastroenterology and Hepatology, Lithuanian University of Health Sciences (LSMU), Kaunas, LTU

**Keywords:** distant metastases, nodal metastases, tumor thickness, metastasis, cutaneous melanoma

## Abstract

Objective

This study aims to analyze melanoma characteristics and their correlation with metastasis in 604 patients diagnosed at the Hospital of Lithuanian University of Health Sciences (2018-2023).

Materials and methods

This retrospective study used coded data from the HLUHS Skin and Venereal Diseases Department database. Data was analyzed using IBM SPSS Statistics; significance was set at p < 0.05.

Results

Nodal metastases were more prevalent in T4 (43.3%) and T3 (29.7%) tumours compared to other thickness groups (p<0.001). Distant metastases increased with tumour thickness (3.0% in T1 to 21.1% in T4) (p<0.001). Ulceration correlated significantly with nodal (32.3% vs. 14.1%) and distant (16.6% vs. 5.7%) metastases (p<0.001). Males exhibit higher rates of advanced stages, nodal and distant metastases, larger tumours, and torso localization; females show higher rates of superficial spreading melanoma and extremity localization.

Conclusions

In Lithuanian cases, T1 melanomas prevailed, but T4 thickness was notably frequent, suggesting potential early detection issues. Tumour thickness correlated with nodal (21.2%) and distant metastases (9.9%), highlighting its predictive significance. Ulceration emerged as a prognostic indicator for distant metastases, with males showing thicker tumours and higher metastasis rates, stressing targeted interventions.

## Introduction

Cutaneous melanomas, characterized by their aggressive nature, are frequently identified in advanced local stages. In 2020, skin melanoma accounted for 4% of new cancer diagnoses and 1.3% of cancer deaths in the European Union (EU-27), making it the sixth most common cancer and one of the top 20 most frequent causes of cancer death [1]. Melanoma is acknowledged as an immunogenic tumour, provoking rapid immune response, and adeptly circumventing immune surveillance through diverse mechanisms. Four distinct histological subtypes of cutaneous malignant melanoma exist, with the superficial spreading subtype prevailing as the most common. Conversely, the nodular subtype emerges as the most aggressive variant, characterized by rapid progression and biologically aggressive behaviour, compared to other histological subtypes of melanoma. For example, in a population-based retrospective study by Allais et al., 18.3% of the patients had nodular, and 81.7% had superficial spreading melanoma. However, the five-year relative survival rate was lower in the group with nodal melanoma compared to superficial spreading (69.0% vs. 98.3%, p < 0.01) [2]. Malignant melanoma can spread via hematogenous or lymphatic pathways, with mortality tied to its high metastatic potential and visceral spread. Melanoma uniquely exhibits locoregional metastasis, presenting as in-transit or satellite metastases, representing disease recurrence. This occurs between the primary tumour and regional lymph nodes in the dermis or subcutaneous tissue, with an incidence of 5-10% and an unfavourable prognosis characterised by low survival rates [3]. The critical prognostic factors encompass the Breslow index, Clark scale, presence of ulceration, microsatellitosis, regression, and lymph node metastasis [4]. The Breslow index retains its position as the primary prognostic factor, shaping disease management in line with recommendations from the National Comprehensive Cancer Network (NCCN) and American Society of Clinical Oncology (ASCO) with studies associating the Breslow index > 4 mm with an increased susceptibility to in-transit metastasis [5,6]. The primary tumour's location could significantly influence locoregional recurrence, as some authors demonstrate a worse prognosis for palm/sole localization and others for the head and neck [7,8]. Understanding the correlation between tumour (T), nodal metastases (N), and distant metastases (M) in melanoma patients is crucial for clinical prognosis assessment. Investigating the impact of primary melanoma location on disease progression and outcome provides insights for risk stratification. Analyzing associations between melanoma types, tumour diameter, and T, N, and M stages enhances prognostication. Our study aims to analyze melanoma characteristics for the Lithuanian population and their correlation with metastasis.

## Materials and methods

Study participants

This retrospective study included 604 patients diagnosed with primary cutaneous melanomas at the Lithuanian University of Health Sciences (HLUHS), between 2018 and 2023. The cases were collected through a retrospective chart review. Initially, electronic medical records (EMRs) and hospital databases were queried using specific diagnostic codes related to primary cutaneous melanomas (e.g., ICD-10 codes for melanoma). Patients included in the study met the inclusion criteria, which required a confirmed diagnosis of primary cutaneous melanoma as documented in their medical records. The medical charts of identified patients were reviewed to confirm the diagnosis and extract relevant clinical data, including demographic information and tumour characteristics. To ensure accuracy, the diagnoses were verified by cross-referencing pathology reports and clinical notes within the EMR system. Initially, 1162 patients with cutaneous melanomas were identified, but 544 were excluded (exclusion criteria involved: undefined diagnosis of melanoma; carcinoma in situ; lentigo maligna; dysplastic nevi; > 1 primary localization of melanoma; basal and squamous cell carcinomas). The patients were included based on the following criteria: having a histopathological diagnosis of cutaneous melanoma; having any type of TNM staging, including Tx, Nx, and Mx; and undergoing a whole-body CT scan after the initial diagnosis to assess the presence of metastases. For all patients, the initial assessment involved dermatoscopic examination, followed by histological confirmation, ensuring accurate T-stage classification. For patients whose melanoma thickness, as per T measurement, exceeded 0.8-1 mm, a sentinel lymph node biopsy (SLNB) was carried out. Those with a positive SLNB underwent PET/CT scans. For individuals with melanoma thickness exceeding 1 mm but with a negative SLNB result, a CT scan of the chest/abdomen/pelvis was performed. Additionally, all patients underwent S100 and lactate dehydrogenase (LDH) blood tests. For those with melanoma thickness below 0.8-1 mm, further assessments included upper abdominal high-resolution ultrasound (UAHRU), chest X-rays, and ultrasound examinations of peripheral lymph nodes.

This study was approved by the Ethics Committee of the Lithuanian University of Health Sciences (HLUHS) (2024-BEC2-370).

Data collection and analysis

**Figure 1:**
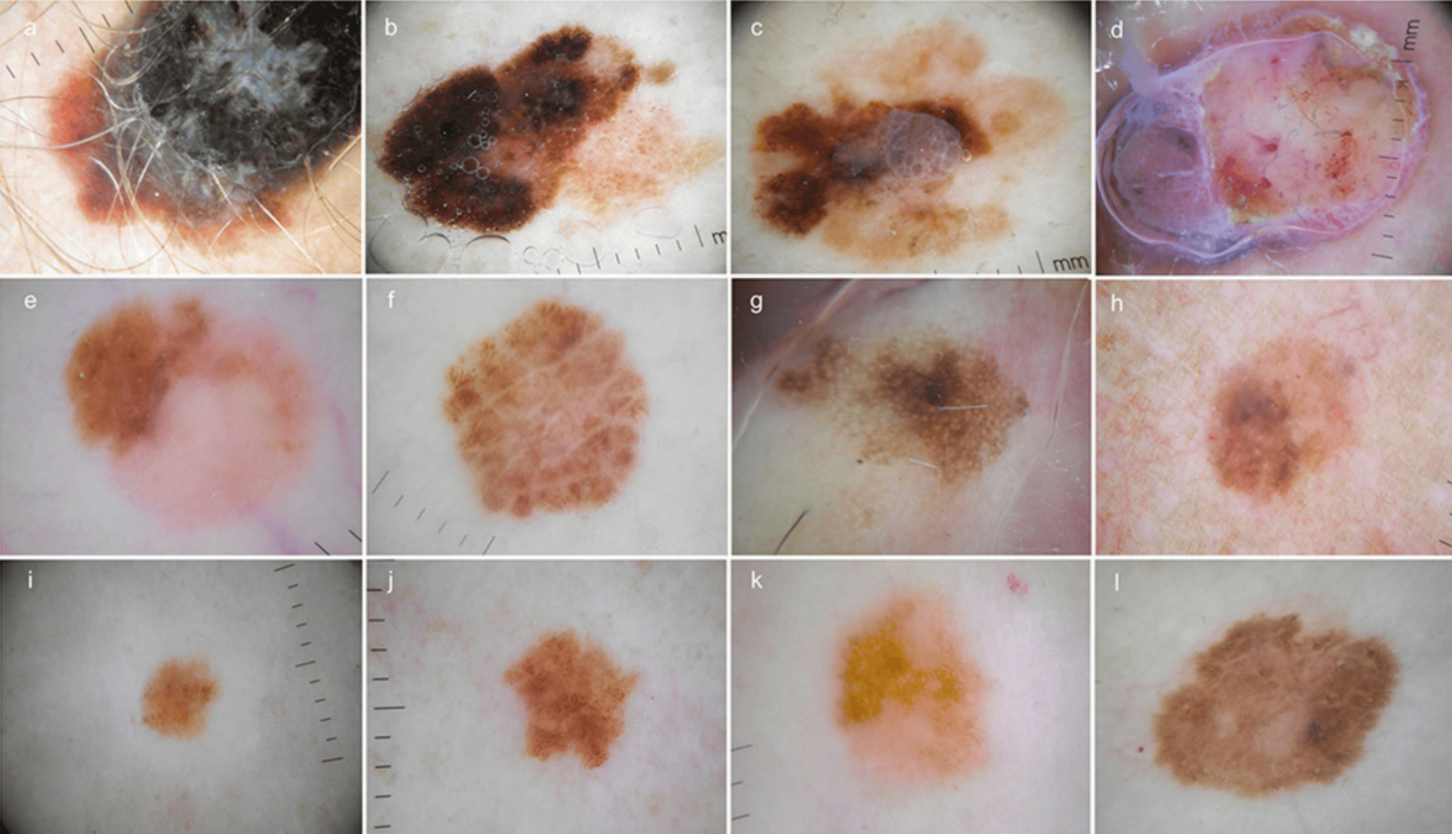
(A) Superficial extensive melanoma, Breslow 2.5 mm; (B) superficial extensive melanoma, Breslow 0.49 mm; (C) superficial extensive melanoma, Breslow 1.7 mm; (D) nodular melanoma, Breslow 3.7 mm; (E) superficial extensive melanoma, Breslow 0.75 mm; (F) superficial extensive melanoma, Breslow 0.35 mm; (G) in situ melanoma; (H) superficial extensive melanoma, Breslow 0.7 mm; (I) in situ melanoma; (J) in situ melanoma; (K) superficial extensive melanoma, Breslow 0.7 mm; (L) superficial extensive melanoma, Breslow 0.6 mm. Copyright: ©2014 Salerni et al. [9]

Patient age, sex, and site of the primary tumour were recorded from the clinical observation papers, and the following features of the primary tumour were extracted from the histopathology reports: histological subtype, presence, or absence of ulceration. The 8th edition of the American Joint Committee on Cancer (AJCC) melanoma staging system was used to assess melanoma characteristics, such as tumour thickness, lymph node and distant metastases. Based on tumour thickness exclusively, we categorised melanomas into four groups: ≤1.0 mm (T1); >1.0-2.0 mm (T2); >2.0-4.0 mm (T3); >4.0 mm (T4). Figure 1 shows dermoscopic images.

Nodal metastases were divided into three groups: no nodal metastases (N0), nodal metastases present (N1-N3), not possible to determine (Nx). Same as with nodal metastases, distant metastases were divided into three groups: no distant metastases (M0), distant metastases present (M1a-d), not possible to determine (Mx). Based on their morphologic type melanomas were categorized into four major groups: superficial spreading; nodular; malignant melanoma (MM), not classified; and others.

Data was analysed using IBM SPSS Statistics (IBM Corp., Armonk, NY, USA). Chi-square and Z-tests were utilised for statistical analysis. Results were considered statistically significant when p < 0.05.

## Results

Baseline characteristics of the study population

A total of 604 subjects were included in the study, 61.4% (n=371) female and 38.6% (n=233) male. The median age of the participants was 64 years (range: 18 to 97). The majority fell within the >60 age group (59.3%, n=358), while the 18-45 age group comprised the smallest percentage at 13.9% (n=84). There was no statistically significant age difference between females and males (p>0.05).

Among the subjects, the most frequent tumour thickness was T1 (n=201; 33.3%), followed by T4 (n=131; 21.7%), T2 (n=101; 16.7%), and T3 (n=171; 28.3%). Nodal metastases were detected in 128 (21.2%) subjects, while 364 (60.3%) had no spreading to the lymph nodes. However, in 112 (18.5%) of the patients, it was not possible to determine whether the tumour had nodal metastases. Distant metastases were found in 60 (9.9%) subjects, while 321 (53.1%) showed no spreading to distant sites. In 223 (37.0%) of the cases, it was impossible to determine whether the tumour had distant metastases. As this study was of retrospective nature, the reason for undetermined lymph node involvement and distant metastases could be only speculated as there was credible data for this discrepancy. Ulceration was present in a substantial number (n=235; 38.9%) of melanoma cases, while the majority (n=369; 61.1%) did not exhibit ulceration. Most commonly ulceration was present in the T4 tumour thickness group (n=127; 21.0%), while in T1 group ulcerated tumours comprised only 6.5% (n=39). In the analysis of melanoma stages among subjects, the breakdown was as follows: stage I (n=292; 48.3%), stage II (n=180; 29.8%), stage III (n=125; 20.7%), and stage IV (n=7; 1.2%).

The most common type of melanoma was superficial spreading melanoma (n=231; 38.2%), while melanomas categorized as “other” (subtypes of melanoma that were not frequent, e.g. Balloon cell melanoma) made up only 8.3% (n=50). It is important to note that a substantial portion of melanomas were not classified, referring to melanoma cases that do not fit neatly into any of the established subtypes of melanoma.

Based on diameter melanomas were categorised into two groups: ≤10 mm and >10 mm. Melanomas with >10 mm diameter were diagnosed most frequently (n=416; 68.7%), while a diameter of ≤10 mm was detected in only 31.3% (n=189) of the subjects. Melanomas among the subjects were located as follows: torso (n=254; 42.1%), lower extremities (n=170; 28.1%), upper extremities (n=110; 18.2%), head and neck (n=70; 11.6%). Table 1 shows the baseline characteristics of the study population.

**Table 1:** Baseline characteristics of the study population.

Melanoma characteristics	N/%
Tumour thickness (T)	
T1 (≤1.0 mm)	201/33.3
T2 (>1.0-2.0 mm)	131/21.7
T3 (>2.0-4.0 mm)	101/16.7
T4 (>4.0 mm)	171/28.3
Nodal metastases (N)	
N0	364/60.3
N1-N3	128/21.2
Nx	112/18.5
Distant metastases (M)	
M0	321/53.1
M1a-d	60/9.9
Mx	223/37.0
Disease stage	
I	292/48.3
II	180/29.8
III	125/20.7
IV	7/1.2
Tumour type	
Superficial spreading	231/38.2
Nodular	153/25.3
Malignant melanoma, not classified	170/28.1
Other	50/8.3
Tumour diameter (mm)	
≤10	189/31.3
>10	415/68.7
Tumour location	
Head, neck	70/11.6
Torso	254/42.1
Upper extremities	110/18.2
Lower extremities	170/28.1
Ulceration	
T1a	162/26.8
T1b	39/6.5
T2a	110/18.2
T2b	21/3.5
T3a	53/8.8
T3b	48/7.9
T4a	44/7.3
T4b	127/21.0

Melanoma characteristics between males and females

When evaluating tumour thickness between female and male subjects, tumours classified as T4 were more prevalent among males (n=86; 36.9%) compared to females (n=85; 22.9%) (p=0.001). Moreover, the female population (n=141; 38.0%) was more likely to be diagnosed with T1 tumours compared to males (n=60; 25.8%) (p=0.001). There was no significant difference between genders in either T2 or T3 groups (p>0.05). The disease stage varied significantly between genders (p<0.001). For example, stage III and IV melanoma were more prevalent in males (n=61; 26.2% and n=6; 2.6%, respectively) compared to females (n=64; 17.3% and n=1; 0.3%, respectively).

It was also determined that the presence of nodal metastases differs significantly between genders (p=0.007). Males showed a higher proportion of nodal metastases (n=64; 27.5%) compared to females (n=64; 17.3%). When analysing the occurrence of distant metastases in different gender groups, it was determined that males have a higher incidence of distant metastases (n=32; 13.7%) compared to females (n=28; 7.5%) (p=0.040). Although, there was no significant difference in the overall status of melanoma ulceration (p>0.05). It should be noted that females (n=112; 30.2%) were more likely to have no ulceration with T1 thickness melanomas compared to males (n=50; 21.5%). Moreover, males (n=69; 29.6%) exhibited a tendency to have ulceration with T4 thickness melanomas more frequently than females (n=58; 15.6%) (p=0.004).

Tumour type also varied significantly between genders (p=0.009). Superficial spreading melanoma was more common in females (n=156; 42.0%), while nodular melanoma more frequent in males (n=74; 31.8%).A statistically significant difference in tumour diameter distribution between different genders was discovered for both categories (≤10 mm and >10 mm) (p<0.001). Larger tumours (>10 mm) were more prevalent among males (n=180; 77.3%) compared to females (n=235; 63.3%). Additionally, female subjects (n=136; 36.7%) tended to have tumours with a diameter of ≤10 mm more frequently than males (n=53; 22.7%). There was a significant difference in tumour location between genders (p<0.001). Males tended to have melanomas more frequently on the torso (n=138; 59.2%) than females (n=116; 31.3%). In contrast, female subjects developed melanomas more frequently on the upper (n=78; 21.0%) and lower (n=136; 36.6%) extremities compared to males (n=32; 13.7% / n=34; 14.6%). Table 2 shows melanoma characteristics between males and females.

**Table 2:** Melanoma characteristics between males and females. * Statistically significant difference (p < 0.05)

Characteristics of melanoma	Gender		
	Female (N/%)	Male (N/%)	
Tumour thickness (T)			
T1 (≤1.0 mm)	141/38.0*	60/25.8*	p=0.001
T2 (>1.0-2.0 mm)	80/21.6	51/21.9	
T3 (>2.0-4.0 mm)	65/17.5	36/15.5	
T4 (>4.0 mm)	85/22.9*	86/36.9*	
Nodal metastases (N)			
N0	230/62.0	134/57.5	p=0.007
N1-N3	64/17.3*	64/27.5*	
Nx	77/20.8	35/15.0	
Distant metastases (M)			
M0	206/55.5	115/49.4	p=0.038
M1a-d	28/7.5*	32/13.7*	
Mx	137/37.0	86/36.9	
Tumour type			
Superficial spreading	156/42.0*	75/32.2*	p=0.009
Nodular	79/21.3*	74/31.8*	
Malignant melanoma, not classified	101/27.2	69/29.6	
Other	35/9.4	15/6.4	
Tumour diameter (mm)			
≤10	136/36.7*	53/22.7*	p<0.001
>10	235/63.3*	180/77.3*	
Tumour location			
Head, neck	41/11.1	29/12.4	p<0.001
Torso	116/31.3*	138/59.2*	
Upper extremities	78/21.0*	32/13.7*	
Lower extremities	136/36.6*	34/14.6*	
Disease stage			
I	199/53.6*	93/39.9*	p<0.001
II	107/28.8	73/31.3	
III	64/17.3*	61/26.2*	
IV	1/0.3*	6/2.6*	
Ulceration			
T1a	112/30.2*	50/21.5*	p=0.004
T1b	29/7.8	10/4.3	
T2a	66/17.8	44/18.9	
T2b	14/3.8	7/3.0	
T3a	33/8.9	20/8.6	
T3b	32/8.6	16/6.9	
T4a	27/7.3	17/7.3	
T4b	58/15.6*	69/29.6*	
With ulceration overall	133/35.8	102/43.8	p=0.052
Without ulceration overall	238/64.2	131/56.2	

The impact of melanoma thickness on metastasis

When evaluating melanoma thickness and its impact on metastasis, the majority of patients with no nodal metastases (N0) were classified as T1 (n=144; 71.6%) and T2 (n=93; 71%). Subjects with tumour thickness classified as T3 (n=55; 54.5%) and T4 (n=72; 42.1%) were less likely to have no lymph node involvement (p<0.001). In contrast, nodal metastases (N1-N3) were observed more often in patients with T4 (n=74; 43.3%) and T3 (n=30; 29.7%) tumour thickness compared to other tumour thickness groups (p<0.001).

Similar findings were observed with distant metastases and tumour thickness. The majority of subjects without distant metastases (M0) were found in the T1 (n=129; 64.2%) and T2 (n=71; 54.2%) tumour thickness groups. Fewer subjects were observed in the T3 (n=51; 50.5%) and T4 (n=70; 40.9%) groups. In contrast, distant metastases (M1a-d) tended to increase with greater tumour thickness. For instance, only 3.0% (n=6) of subjects with T1 had distant metastases, whereas 21.1% (n=36) of those with T4 had them (p<0.001). Table 3 shows the impact of melanoma thickness on metastasis.

**Table 3:** Impact of melanoma thickness on metastasis. * Statistically significant difference (p < 0.05)

Characteristics of melanoma	Tumour thickness (N/%)				
	T1 (≤1.0 mm)	T2 (>1.0-2.0 mm)	T3 (>2.0-4.0 mm)	T4 (>4.0 mm)	
Nodal metastases (N)					
N0	144/71.6*	93/71*	55/54.5*	72/42.1*	p<0.001
N1-N3	7/3.5*	17/13.0*	30/29.7*	74/43.3*	
Nx	50/24.9*	21/16.0	16/15.8	25/14.6*	
Distant metastases (M)					
M0	129/64.2*	71/54.2*	51/50.5*	70/40.9*	p<0.001
M1a-d	6/3.0*	8/6.1*	10/9.9*	36/21.1*	
Mx	66/32.8	52/39.7	40/39.6	65/38.0	

Distribution of tumour thickness and metastases in different anatomical locations

In our evaluation, we found that the distribution of tumour thickness and nodal metastases varied across different anatomical locations. However, there was no significant difference observed when evaluating the distribution of distant metastases in different tumour locations (p>0.05).

T1 tumours were most commonly diagnosed in subjects with tumour locations on the torso (n=100; 39.4%) and least frequently in the head and neck (n=14; 20.0%), with a significant difference between these two groups (p=0.004). T3 tumours were more prevalent in those with tumours on the head and neck (n=20; 28.6%) and upper extremities (n=23; 20.9%), than on the torso (n=28; 11.0%) (p=0.004). There was no significant difference observed in the T2 and T4 groups (p>0.05). The absence of lymph node involvement was most common in patients with tumours on the lower extremities (n=109; 64.1%), and least common on the head and neck (n=35; 50.0%), with a significant difference between these two groups (p=0.001). Nodal metastases were most commonly observed in torso tumours (n=63; 24.8%), while they were least frequent in head and neck tumours (n=10; 14.3%) and upper extremity tumours (n=17; 15.5%). Tumours on the torso were significantly more likely to have metastases compared to those on the upper extremities (p=0.001). However, due to a high proportion of individuals with undetermined nodal and distant metastases, these statistical conclusions are likely unreliable. Table 4 shows the distribution of tumour thickness and metastases in different anatomical locations.

**Table 4:** Distribution of tumour thickness and metastases in different anatomical locations. * Statistically significant difference (p < 0.05)

Characteristics of melanoma	Location (N/%)					
	Head, neck	Torso	Upper extremities		Lower extremities	
Tumour thickness (T)						
T1 (≤1.0 mm)	14/20.0*	100/39.4*		37/33.6*	50/29.4*	p=0.004
T2 (>1.0-2.0 mm)	15/21.4	47/18.5		26/23.6	43/25.3	
T3 (>2.0-4.0 mm)	20/28.6*	28/11.0*		23/20.9*	30/17.6	
T4 (>4.0 mm)	21/30.0	79/31.1		24/21.8	47/27.6	
Nodal metastases (N)						
N0	35/50.0*	153/60.2		67/60.9	109/64.1*	p=0.001
N1-N3	10/14.3	63/24.8*		17/15.5*	38/22.4	
Nx	25/35.7*	38/15.0*		26/23.6*	23/13.5*	
Distant metastases (M)						
M0	35/50.0	131/51.6		59/53.6	96/56.5	p=0.791
M1a-d	6/8.6	29/11.4		8/7.3	17/10.0	
Mx	29/41.4	94/37.0		43/39.1	57/33.5	

The distribution of melanoma ulceration and its influence on metastasis

There is a significant association between ulceration status and the presence of nodal metastases (p < 0.001).* *A higher percentage of cases with ulceration (n=76; 32.3%) have nodal metastases compared to those without ulceration (n=52; 14.1%). Similarly, for distant metastases, there is a significant association between ulceration status and the presence of distant metastases (p < 0.001). A higher percentage of cases with ulceration (n=39; 16.6%) have distant metastases compared to those without ulceration (n=21; 5.7%). In both cases, the absence of nodal or distant metastases was more frequent in those patients with no tumour ulceration (p<0.001). These findings suggest that melanomas with ulceration are more likely to be associated with both nodal and distant metastases compared to melanomas without ulceration. Table 5 shows the distribution of melanoma ulceration and its influence on metastasis.

**Table 5:** Distribution of melanoma ulceration and its influence on metastasis. * Statistically significant difference (p < 0.05)

Characteristics of melanoma	Ulceration (N/%)		
	Without	With	
Nodal metastases (N)			
N0	243/65.9*	121/51.5*	p<0.001
N1-N3	52/14.1*	76/32.3*	
Nx	74/20.1	38/16.2	
Distant metastases (M)			
M0	210/56.9*	111/47.2*	p<0.001
M1a-d	21/5.7*	39/16.6*	
Mx	138/37.4	85/36.2	

When evaluating the impact of ulceration on nodal and distant metastasis within the same tumour thickness groups, no significant difference was found (p > 0.005).

## Discussion

The majority of melanomas in our study population were categorised as T1, however, the second most common tumour thickness was T4. This distribution suggests a gap in early tumour detection, as some individuals are diagnosed early, while others present with a more advanced disease. Nodal metastases were detected in 21.2% of the subjects, while distant metastases were found in 9.9%. It is important to mention that in a substantial number of cases in our study population, it was not possible to determine the presence of nodal or distant metastases, which may indicate limitations in diagnostic techniques or incomplete data. This may have resulted from incomplete patient records, leading to gaps in the data. Moreover, differences in follow-up duration and consistency among patients could have affected the detection of metastases, leading to variability in the data. To compare, in a study conducted in Eastern Finland, Suhonen et al. report a lower percentage of metastasis by ~10% in their population (lymph nodes - 15.7%, and/or distal organs - 12.8%) [10]. In a nationwide cohort study with the Swedish population, Zheng et al. outlined that 48.0% of the patients were diagnosed with T1a, followed by T2a (16.3%) [11].

In our study, stage I was the most common (48.3%), followed by stage II (29.8%), stage III (20.7%), and stage IV (1.2%). Although, this distribution highlights a predominance of early-stage melanomas, a prominent portion of individuals were diagnosed with later stages of melanoma, particularly stage III. Rockberg et al. report that the majority (92.0%) of patients diagnosed with a first melanoma diagnosis in Sweden have localised disease (stages I and II), while stages III and IV were present in only 8.0% of the patients [12]. Robsahm et al. outlined that 91.8% of the Norwegian cases were diagnosed at a local stage, whereas 5% had regional metastases and 3.2% had distant metastases [13].

When assessing the impact of melanoma thickness on nodal metastasis in our study population, patients with thicker tumours (T3 and T4), were less probable to exhibit an absence of lymph node involvement (N0) compared to those with thinner tumours (T1 and T2) (p<0.001). Conversely, nodal metastases (N1-N3) were more commonly observed in patients with T3 and T4 (p<0.001). Moy et al. found that patients with primary cutaneous melanoma without lymphatic invasion were more likely to be melanoma-free at the last follow-up compared to those with lymphatic invasion (80% vs. 50%, respectively; p=0.002) [14]. A similar trend was observed with distant metastases, while T1 and T2 tumours were predominantly associated with an absence of distant spread (M0), a progression in tumour thickness correlated with an increase in distant metastasis. Moy et al. discovered via single-variable analysis that tumour thickness was significantly correlated with melanoma progression (p=<0.001) [14]. These results showcase that as tumour thickness increases, so does the frequency of both nodal and distant metastases. This is important in predicting the likelihood of nodal and distant metastases in melanoma patients across different melanoma stages.

Our study reinforces the established idea that ulceration is a crucial prognostic marker in melanoma, as we observed a notable increase in nodal and distant metastasis among cases with ulceration compared to those without such lesions. Robsahm et al. published similar results, indicating that ulceration increased with tumour thickness and was present in 73.8% of T4 tumours in Norwegian patients diagnosed with cutaneous melanoma [13]. Similarly in our cohort, patients with ulcerated melanomas had a significantly higher likelihood of distant metastases compared to those without (p < 0.001). Moy et al.’s univariate analysis revealed that the presence of ulceration was significantly associated with disease progression (p=0.017) [14]. This suggests that ulcerated melanomas are more prone to systemic spread, leading to distant metastases in organs or tissues beyond the primary site, indicating a more aggressive disease behaviour.

When evaluating melanoma characteristics between males and females, the male gender was associated with negative prognostic attributes for melanoma, such as tumour thickness, nodal and distant metastasis, late stage (III and IV), nodular type and greater diameter of melanoma (p < 0.05). Similar findings could be found in other studies, examining melanoma characteristics between different genders. Suhonen et al. report that men exhibit a higher metastasis rate compared to women (28.5% vs. 13.9%, p = 0.001) [10]. In a study by Robsahm et al. [13], men tended to have thicker and more invasive tumours than women. In women, 56% of the tumours were stage I, while in men the proportion was 47.6%. Zheng et al. noted that female patients exhibited a more favourable prognosis in comparison to males (0.69, 95% CI: 0.64-0.74) [11]. The distribution of melanoma type also differed between genders, with superficial spreading melanoma being more common in females (70.6% in women and 65.8% in men), while nodular melanoma occurred more frequently in males (22.8% in women and 28.6% in men) [10]. Our study also revealed a significant difference in tumour location between genders (p<0.001), as males (59.2%) had a higher prevalence of melanomas on the torso than females (31.3%), while females had a higher frequency of melanomas in the upper (21.0%) and lower (36.6%) extremities compared to males (13.7% / 14.6%). The tendency of tumour location between genders has been described in other studies. Suhonen et al. found that the prevalence of TANS (thorax, upper arm, neck, and scalp) melanomas was significantly higher in males compared to females, as well as melanomas of the trunk (p < 0.001). Females exhibited a higher incidence of head and neck (p = 0.007) and lower limb melanomas compared to males (p < 0.001) [10]. Robsahm et al. reported that the trunk was the most common location (48.0%) in Norwegian patients, but it was more common in men (60.4%) than in women (35.8%) [13]. It may be hypothesised that tumour location differences may be attributed to different clothing choices between genders, influencing the areas of the body exposed to sunlight. It could be hypothesised that sun exposure habits, influenced by outdoor activities, and sun protection practices, might contribute to the observed differences in melanoma characteristics between Lithuanian men and women. Additionally, cultural norms and clothing choices may differ between genders, influencing the areas of the body exposed to sunlight and contributing to gender-specific disparities in melanoma presentation. Disparities in healthcare-seeking behaviour further impact the stage at which melanoma is diagnosed, as females tend to exhibit more proactive tendencies, while males often delay seeking medical advice until the disease has progressed. Overall, these results of social factors may shape gender differences in melanoma characteristics, with males presenting with more adverse prognostic features.

Study limitations

Considering the limitations of the study, retrospective studies are subject to selection bias, where the study population might not accurately represent the general population or the intended sample. In a substantial number of cases, it was not possible to determine the presence of nodal or distant metastases. Variability in the quality and accuracy of diagnostic techniques used across different periods and differences in follow-up duration and consistency among patients could have affected the consistency of metastasis detection. This may have resulted from incomplete patient records, leading to gaps in the data. Another limitation of our study is the unavailability of comprehensive medical histories for the included patients, which would have been valuable for obtaining a more thorough information about the study population. Moreover, this study could be improved by including a larger and more representative study population to enhance the statistical power of findings.

## Conclusions

Our study provides a detailed overview of melanoma characteristics in Lithuania, revealing significant variations when compared to global data. We found a predominant occurrence of early-stage melanomas, particularly T1, alongside a notable presence of T4 tumours, highlighting challenges in early detection and varying disease presentation within our population. Nodal metastases were detected in 21.2% of cases, with distant metastases in 9.9%, although incomplete data in some cases suggested diagnostic limitations or record-keeping issues. Comparisons with global epidemiological trends underscored lower metastasis rates in Eastern Finland and Sweden, contrasting with our findings and indicating distinct disease progression patterns influenced by regional healthcare practices. Gender disparities were evident, with males exhibiting higher rates of thicker tumours, nodal metastases, and advanced stages compared to females, aligning with global patterns. Ulceration emerged as a critical prognostic factor, significantly correlating with increased nodal and distant metastases, reinforcing its importance in clinical management strategies. Understanding these regional variations is crucial for refining melanoma prevention, early detection efforts, and treatment strategies in Lithuania.
